# Computational screen to identify potential targets for immunotherapeutic identification and removal of senescence cells

**DOI:** 10.1111/acel.13809

**Published:** 2023-04-20

**Authors:** Eden Z. Deng, Reid H. Fleishman, Zhuorui Xie, Giacomo B. Marino, Daniel J. B. Clarke, Avi Ma'ayan

**Affiliations:** ^1^ Department of Pharmacological Sciences, Mount Sinai Center for Bioinformatics Icahn School of Medicine at Mount Sinai New York New York USA

**Keywords:** antibody drug conjugates, bioinforatics, biomarkers, CAR T‐cell therapy, neoantigens, omics integration

## Abstract

To prioritize gene and protein candidates that may enable the selective identification and removal of senescent cells, we compared gene expression signatures from replicative senescent cells to transcriptomics and proteomics atlases of normal human tissues and cell types. RNA‐seq samples from in vitro senescent cells (6 studies, 13 conditions) were analyzed for identifying targets at the gene and transcript levels that are highly expressed in senescent cells compared to their expression in normal human tissues and cell types. A gene set made of 301 genes called SenoRanger was established based on consensus analysis across studies and backgrounds. Of the identified senescence‐associated targets, 29% of the genes in SenoRanger are also highly differentially expressed in aged tissues from GTEx. The SenoRanger gene set includes previously known as well as novel senescence‐associated genes. Pathway analysis that connected the SenoRanger genes to their functional annotations confirms their potential role in several aging and senescence‐related processes. Overall, SenoRanger provides solid hypotheses about potentially useful targets for identifying and removing senescence cells.

## Graphical abstract

Data from published studies of senescence cells were used to identify genes and transcripts that are commonly upregulated in these cells compared to their expression levels across normal tissues and cell types processed from GTEx, ARCHS4, and Tabula Sapiens. Identified genes and transcripts were further filtered to include only genes and transcripts that give rise to membrane proteins and secreted proteins. These genes and transcripts may serve as useful targets for identifying and removing senescence cells.

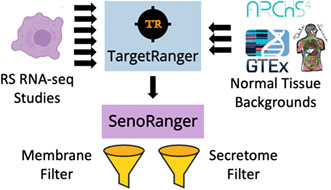

Cellular senescence is a state of permanent cell cycle arrest that occurs in somatic cells. Cellular senescence is a tightly regulated biological process in normal physiology (Campisi & d'Adda di Fagagna, 2007). However, senescence has also been implicated as a key process in aging, where senescent cells avoid clearance by the immune systems and accumulate to contribute to age‐related pathologies (Childs et al., 2015; van Deursen, 2014). In some mouse tissues such as liver, spleen, skin, and lung, it is estimated that the presence of senescence cells increases from ~3%–5% in young tissues to 20%–30% in old tissues (Wang et al., 2009). Indeed, clearance of senescent cells has been demonstrated to delay the onset of aging phenotypes in progeroid mouse models (Baker et al., 2011) and increased metabolic function (Xu et al., 2018) and healthy lifespan (Childs et al., 2015) in naturally aged mice. As targeted removal of senescent cells gains more research interest, there is a need for identifying targets for potential removal of various senescent cell types. Here we processed background normal tissue and cell type datasets from several gene and protein atlases, and developed software called TargetRanger (http://targetranger.maayanlab.cloud) to identify genes and proteins that are uniquely highly expressed in senescence cells for targeting senescent cells. The results of such analysis are ranked lists of potential targets for senolytic therapy. To identify a set of cell surface candidates for immunotherapeutic removal of senescent cells, we sequentially filtered highly expressed senescence genes in different contexts as follows. First, highly expressed genes were obtained from six independent studies of replicative senescence (RS) (Casella et al., 2019; De Cecco et al., 2019; Marthandan et al., 2015, 2016; Sen et al., 2019; Voic et al., 2019) (Table S1). From these studies, we identified 13 groups of samples that are considered representative of RS. Next, we compared these 13 groups to gene expression profiles of normal tissues and cell types processed from GTEx (Lonsdale et al., 2013), ARCHS4 (Lachmann et al., 2018), and Tabula Sapiens (The Tabula Sapiens, Consortium, 2022). Candidates were considered significantly highly expressed genes in the RS groups if they are lowly expressed across all normal tissues and cell types from these resources. Overall, 39 comparisons were made at the gene level (GTEx, ARCHS4, and Tabula Sapiens), and 26 comparisons were made at the transcript level (GTEx and ARCHS4). Significantly differentially expressed candidates were harvested from all comparisons to identify the most consistent candidates. In addition, the consensus candidates were filtered to only include genes that give rise to at least one of 2054 membrane‐bound proteins, and 1536 secreted proteins, obtained from COMPARTMENTS (Binder et al., 2014) and the human protein atlas (HPA) (Rozenblatt‐Rosen et al., 2017), respectively. Next, we compared the identified targets to genes highly differentially expressed in aged and age‐associated diseased tissues. Genes significantly highly expressed in aged versus younger tissues were obtained from Chatsirisupachai et al. (2019) for 13 different tissue types, and genes highly expressed in senescence‐mediated diseases were obtained from an idiopathic pulmonary fibrosis study (Schafer et al., 2017), and a heart failure study (Tarazón et al., 2014). Identified targets were also compared to normal protein expression levels obtained from three proteomics atlases, namely HPA (Uhlén et al., 2015), human proteome map (HPM) (Kim et al., 2014), and GTEx proteomics (Jiang et al., 2020). Potential targets from this analysis were noted for differential expression in aging tissues, diseased tissues, and whether they are highly expressed at the protein level. The overall pipeline is visually presented as a workflow diagram (Figure 1a). From the significantly differentially expressed gene targets, across all analysis conditions, we identified 301 genes and 1058 transcripts (Table S2) that are significantly highly expressed in senescence cells for at least 25% of the analysis conditions. Of the 301 senescence genes, 38 encode cell surface proteins (Table S3, Figure 1b), and 83 encode secreted proteins (Table S4, Figure 1c). A significant portion of these proteins were also observed at the transcript level.

**FIGURE 1:**
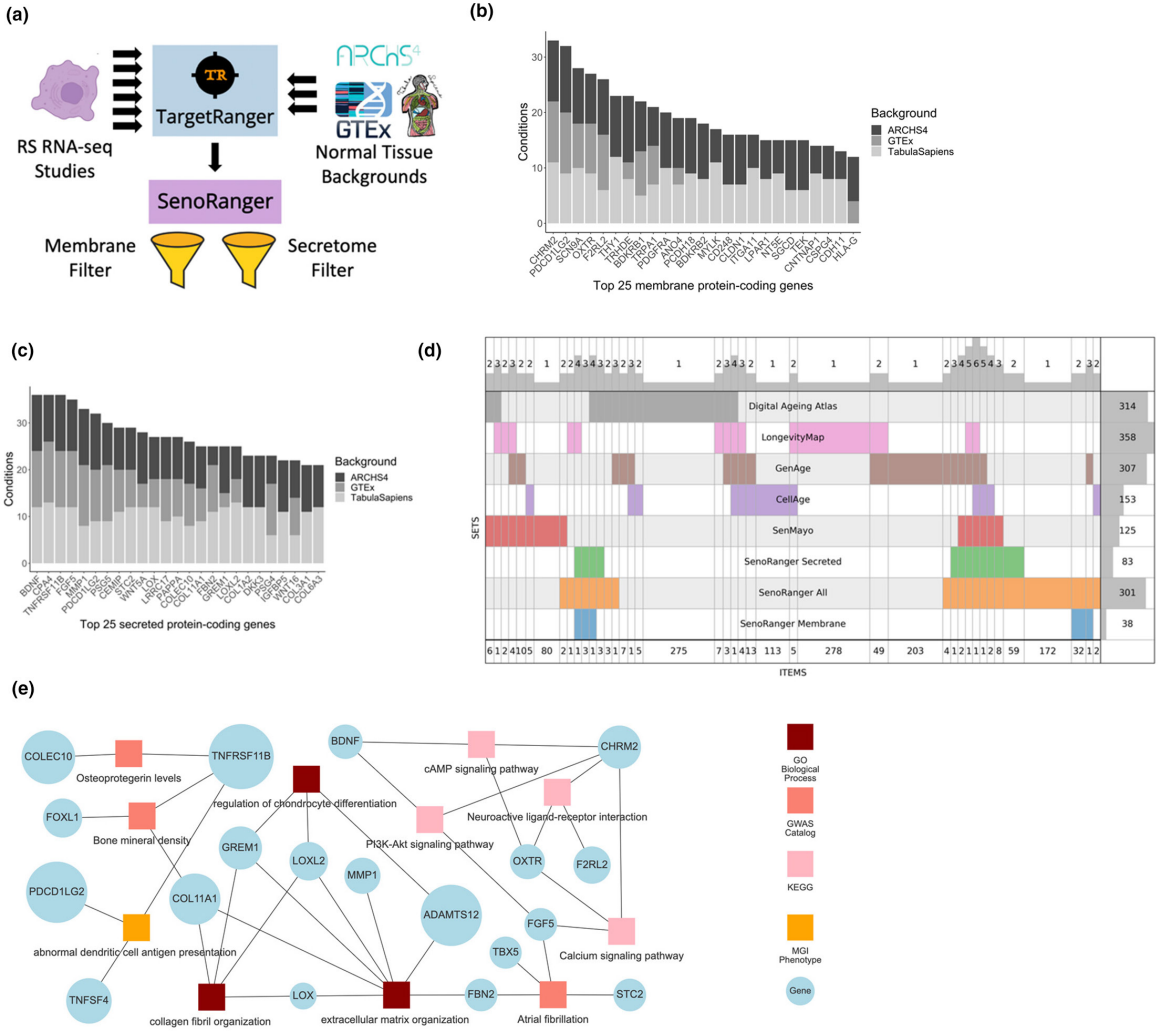
(a) The SenoRanger workflow. Data from published studies of senescence cells were used to identify genes that are commonly upregulated in these cells compared to their expression levels across normal tissues and cell types processed from GTEx, ARCHS4, and Tabula Sapiens. Identified genes were further filtered to include only genes and transcripts that give rise to membrane proteins and secreted proteins. The top selected genes and transcripts are also compared to genes and transcripts that are highly expressed in aging tissues and previously published gene sets related aging and senescence. (b) Top 25 most consistent transmembrane targets identified by TargetRanger. (c) Top 25 most consistent secreted targets identified by TargetRanger. (d) SuperVenn diagram comparing the SenoRanger genes to gene sets from the Digital Aging Atlas, Longevity Map, GeneAge, CellAge, and SenMayo. (e) The most significant enriched terms from the Gene Ontology Biological Processes, GWAS Catalog, KEGG pathways, and MGI phenotypes gene set libraries in Enrichr connected to genes from SenoRanger.

The final lists of 301 genes, which we termed SenoRanger, the 38 cell surface proteins, and the 83 secreted proteins were further compared to gene sets extracted from LongevityMap (Tacutu et al., 2018), CellAge (Tacutu et al., 2018), Digital Ageing Atlas (Craig et al., 2015), GenAge (Tacutu et al., 2018), and SenMayo (Saul et al., 2022). The results from such comparisons are visualized as a SuperVenn diagram (Figure 1d) and as UMAP plots (Figure S1). This overlap analysis suggests that the SenoRanger list shares similarity with those previously published senescence and aging gene sets. In addition, the UMAP plot shows that the GTEx background produces gene sets that have less in common with previously published senescence and aging gene sets, and some of the RS studies included are potentially less relevant compared with others. It is surprising that the ARCHS4 and the Tabula Sapiens backgrounds produced similar results.

To observe whether any cell‐surface and secreted protein targets are also highly expressed in aged and/or diseased tissues with known senescence markers, we compared the SenoRanger gene set to genes enriched in aged tissues, idiopathic pulmonary fibrosis, and heart failure. RNA‐seq gene counts from lung tissue (GSE92592) of idiopathic pulmonary fibrosis patients (*N* = 20) compared to healthy controls (*N* = 19) (Schafer et al., 2017), as well as from left ventricular tissue (GSE55296) of patients with heart failure (*N* = 26) compared to healthy controls (*N* = 10) (Tarazón et al., 2014) were processed using TargetRanger to identify genes significantly highly expressed in the diseased tissues where senescent cells are known to accumulate. Of the 301 SenoRanger genes, 28.9% (*n* = 87) were also highly expressed in older versus younger GTEx adipose, adrenal, blood vessel, brain, colon, heart, liver, lung, muscle, nerve, pancreas, skin, or thyroid tissues, and 10% (*n* = 30) were highly expressed in IPF versus control lung tissues, or heart failure versus control left ventricular tissues (Table S2).

Enrichr analysis of these genes (Kuleshov et al., 2016) identified significant phenotypes such as reduced bone mineral density (GWAS Catalog) (MacArthur et al., 2017) and abnormal immune response (MGI Mammalian Phenotypes) (Smith & Eppig, 2009) (Figure 1e). Using drug signatures computed from the Gene Expression Omnibus (GEO) database, the common senescence genes were significantly enriched for genes downregulated with metformin (a diabetic drug that alters cell metabolism) and genes upregulated with cisplatin (chemotherapy drug). Notably, the SenoRanger gene set was enriched for genes downregulated with overexpression of JAG1, a NOTCH1 ligand whose decrease in expression with age may reduce Notch signaling and senescent cell clearance in human skin (Yoshioka et al., 2021). The SenoRanger genes were also associated with a variety of transcription factors, including CBX2 (ENCODE) which was found to be an essential regulator of senescence‐associated chromosomal instability in mouse fibroblasts (Baumann et al., 2020).

To make the computational screening of targets from RNA‐seq samples collected from cells that may be targeted for identification and removal, we developed the TargetRanger. TargetRanger is web‐based software application available from https://targetranger.maayanlab.cloud. It is free to use without log in requirements. Users of TargetRanger need to upload processed RNA‐seq samples in a specified format, select the background and filters, and then, after pressing the submit button, they are presented with plots and tables of top ranked potential targets. The approach can be applied to other disease conditions such as identifying targets for the identification and removal of solid tumors. In conclusion, SenoRanger suggests potential targets for identifying and removing senescence cells. However, experimental validation that considers tissue specificity and dosing will be required to enable practical applications.

## AUTHOR CONTRIBUTIONS

Eden Z. Deng prepared the RS expression data from GEO and the proteomics data from HPM, HPA and GTEx proteomics, performed the analysis, generated the figures, and wrote portions of the manuscript. Reid H. Fleishman processed the data from Tabula Sapiens. Zhuorui Xie performed the set overlap analysis. Giacomo B. Marino contributed to the development of the TargetRanger web application. Daniel J. B. Clarke developed the computational framework to perform the analysis and prepared the GTEx and ARCHS4 transcriptomics background. Avi Ma’ayan wrote the paper, conceptualized the study, and supervised the project.

## CONFLICT OF INTEREST STATEMENT

The authors declare that they do not have any conflicts of interests.

## Supporting information


**Supporting information S1.** Supplementary material.

**Table S2** Top consensus genes identified to be highly expressed in replicative senescence cells compared to normal tissues and cell type backgrounds.

**Table S3** Top consensus membrane proteins identified to be highly expressed in replicative senescence cells compared to normal tissues and cell type backgrounds.

**Table S4** Top consensus secreted proteins identified to be highly expressed in replicative senescence cells compared to normal tissues and cell type backgrounds.

## Data Availability

All code and processed data are available from https://github.com/MaayanLab/SenoRanger.
