# Supplementary material for: Computational screen to identify potential targets for immunotherapeutic identification and removal of senescence cells
Source: Aging Cell. 2023 Apr 20;22(6):e13809. doi: 10.1111/acel.13809 (PMC10265163; doi:10.1111/acel.13809)
Supplement: Supplementary file 1 — Supporting information S1. Supplementary material. [file ACEL-22-e13809-s001.docx]

# **Supporting Online Materials for:**

# Computational Screen to Identify Potential Targets for Immunotherapeutic Identification and Removal of Senescence Cells

Eden Z. Deng^1^, Reid H. Fleishman^1^, Xie, Zhuorui^1^, Giacomo B. Marino^1^, Daniel J. B. Clarke^1^, Avi Ma’ayan^1,*^

# ^1^Department of Pharmacological Sciences, Mount Sinai Center for Bioinformatics, Icahn School of Medicine at Mount Sinai, One Gustave L. Levy Place, Box 1603, New York, NY 10029, USA

*To whom correspondence should be addressed: [avi.maayan@mssm.edu](mailto:avi.maayan@mssm.edu)

# **Supporting Figures**

# Fig. S1

# A


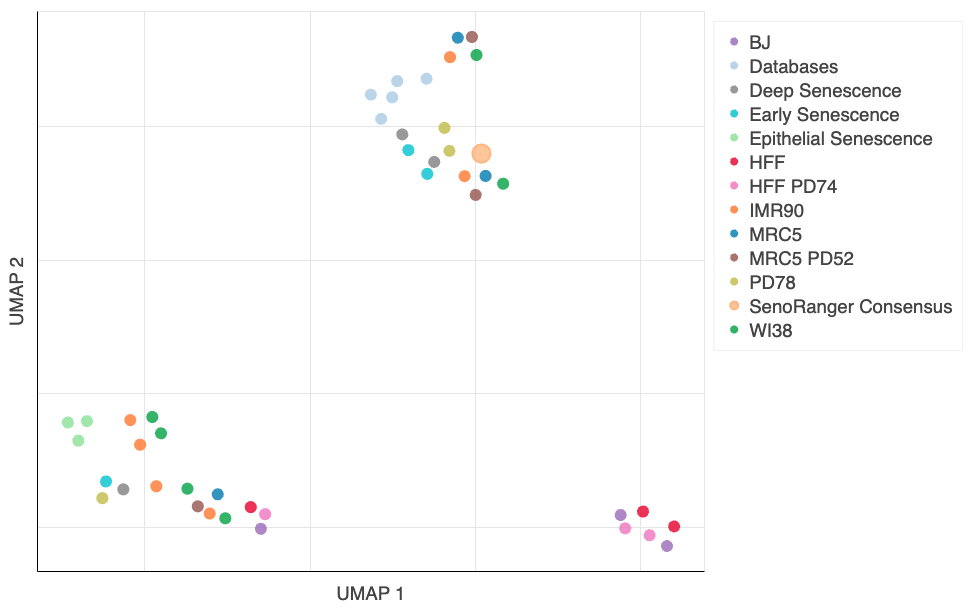


# B


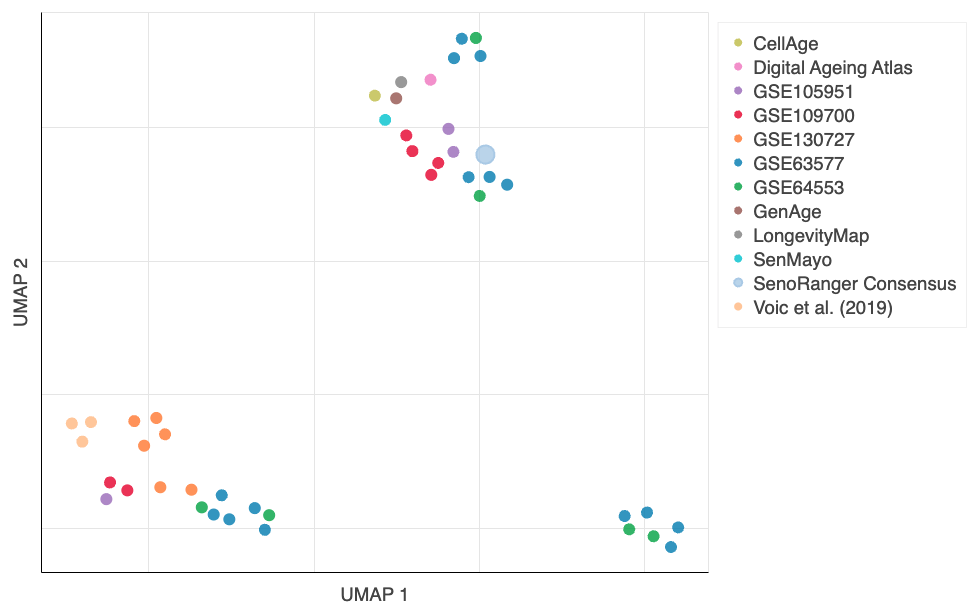


# C


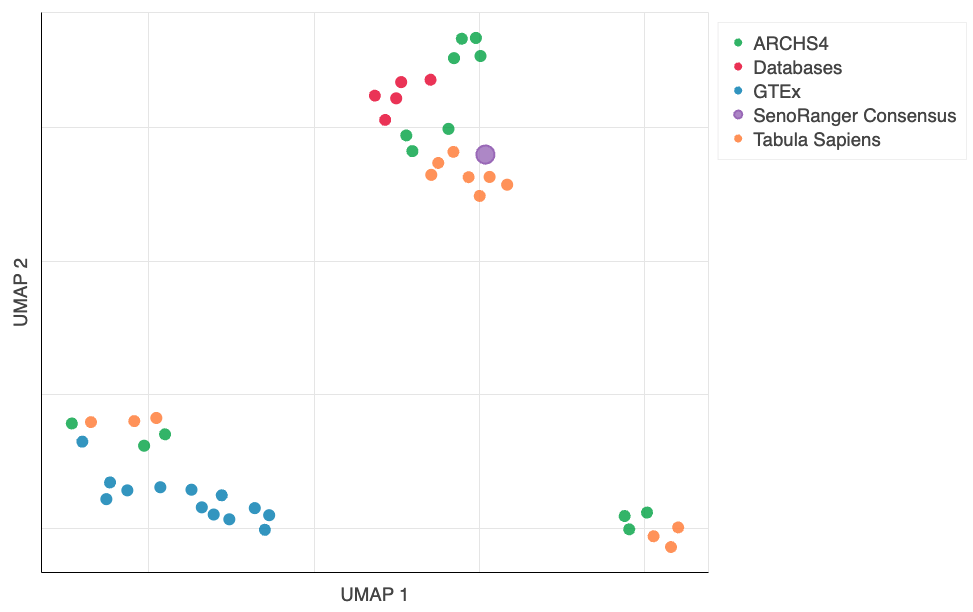


# **Fig. S1 UMAP visualization of gene similarity by comparing gene sets identified in this study to previously published senescence and aging related gene sets.** A) Gene sets are colored by the cell line or cell type used in the original replicative senescence study. B) Gene sets are colored by the original study used to generate the gene set. C) Gene sets are colored by the normal tissue background used to produce the gene set. The consensus SenoRanger gene set is highlighted in a larger circle across all three panels.

# **Supporting Tables**

| **Study Author(s)** | **Study Title** | **Cell Types and Samples** |
| --- | --- | --- |
| Marthandan et al. (2015) | Hormetic effect of rotenone in primary human fibroblasts | WI-38 fibroblasts (n = 3)  IMR-90 fibroblasts (n = 3) |
| Marthandan et al. (2016) | Conserved senescence associated genes and pathways in primary human fibroblasts detected by RNA-seq | MRC-5 fibroblasts (n = 3)  WI-38 fibroblasts (n = 3)  BJ fibroblasts (n = 3)  IMR-90 fibroblasts (n = 3)  HFF fibroblasts (n = 3) |
| Casella et al. (2019) | Transcriptome signature of cellular senescence | WI-38 fibroblasts (n = 2)  IMR-90 fibroblasts (n = 2) |
| DeCecco et al. (2019) | L1 drives IFN in senescent cells and promotes age-associated inflammation | LF1 fibroblasts - early senescence (n = 3)  LF1 fibroblasts - deep senescence (n = 3) |
| Sen et al. (2019) | Histone acetyltransferase p300 induces de novo super-enhancers to drive cellular senescence | IMR-90 fibroblasts (n = 4) |
| Voic et al. (2019) | RNA sequencing identifies common pathways between cigarette smoke exposure and replicative senescence in human airway epithelia | Bronchial epithelial cells (n = 5) |

**Table S1 Senescence studies and RNA-seq samples**

**Table S2 Top consensus genes identified to be highly expressed in replicative senescence cells compared to normal tissues and cell type backgrounds**

**Table S3 Top consensus membrane genes identified to be highly expressed in replicative senescence cells compared to normal tissues and cell type backgrounds**

**Table S4 Top consensus secreted genes identified to be highly expressed in replicative senescence cells compared to normal tissues and cell type backgrounds**

# **Supporting Methods**

*Processing RNA-seq Data from Replicative Senescence Cells:* Senescent cell RNA-seq datasets were obtained from six published studies (12,39-43) (Table 1). These studies performed RNA-sequencing of cells serially-passaged into replicative senescence (RS). In total, we included 40 RS samples collected from across 6 different cell types, comprising 13 conditions. Gene counts from each study were downloaded by cell type from GEO, or directly from the publication. Transcript counts were processed from the raw FASTQ files using the Elysium: cloud alignment tool (44).

*Processing the 10x Genomics Data from Tabula Sapiens:* The Tabula Sapiens dataset used for this study was retrieved on May 4th, 2022, Version 4 release from Figshare. The file that was downloaded is called TabulaSapiens.h5ad.zip (14.51 GB). The AnnData .h5ad file was extracted from the ZIP file and made into an AnnData object in Python. The raw Tabula Sapiens data was then filtered to only include cells processed with any 10x Genomics method. Then, the AnnData file was split into each donor using the “donor” observation. For each donor, a sparse matrix was extracted from the “decontXcounts” decontaminated counts layer, the gene symbols were extracted from the “gene_symbol” observation, and the cell type labels were extracted from the “cell_ontology_class” observation. The name of each cell’s tissue of origin was extracted from the “organ_tissue” observation and combined with the cell type labels. A sparse Pandas dataframe was then created with this data with gene symbols as the rows and tissue-cell types as the columns. To create pseudo-bulk RNA-seq data from the scRNA-seq data, we summed the counts of each cell type. This process was repeated for each donor, and the final dataframe was a concatenation of all processed data frames from the donors. For each tissue-cell type dataframe, the dataframe was transposed and grouped by tissue-cell type to generate statistics over the tissue-cell types for each gene. These statistics were count, mean, std, min, 25% quartile, 50% quartile, 75% quartile, and max; these were generated over the non-zero counts.

*Processing RNA-seq Data from GTEx:* To create the GTEx normal tissue background, RNA-seq gene and transcript counts from donors ages 20-79 were obtained from the Genotype-Tissue Expression (GTEx) consortium data portal (version 8 dataset) (45), covering 47 tissue types. The 1st quartile, median, and 3rd quartile of each gene and transcript raw counts were computed for each tissue type.

*Processing RNA-seq Data from ARCHS4:* To create the normal tissue gene expression background from ARCHS4 (46), GEO sample descriptions were first tokenized. Tokens were then manually annotated to capture disease, tissue, cell type, control, drug, and cell line terms. Using ARCHS4's single cell probability attribute, samples originated from single cell RNA-seq datasets were filtered out. Using each set of GEO samples corresponding to each tissue type, cell type, or cell line, descriptive statistics of the raw expression counts for each gene were computed. These statistics include min, max, mean, standard deviation, and count along with the first, second (median) and third quartiles. Differential gene expression was computed against the first, second, and third quartiles of each tissue, cell type, and cell line. The ARCHS4 v11 dataset was used for this data processing step.

*Processing proteomics data from HPA, HPM, and GTEx:* Protein expression levels from normal human tissues and cell types were obtained from three resources: the Human Protein Atlas (HPA) (47), the Human Proteome Map (HPM) (48), and the GTEx proteomics project (49). The HPA provides protein expression at four discrete levels (not detected, low, medium, high) across 44 different tissue types from 3 donors, covering 15,323 genes. Profiling of protein expression in each tissue was based on immunohistochemistry (IHC) staining of the tissue and knowledge-based annotation of the images. Protein expression levels and reliability scores were reported by gene name, based on HPA version 21.1 and retrieved from the HPA portal. HPM provides quantitative measures of protein expression from 3 donors across 17 adult tissues, 7 fetal tissues, and 6 hematopoietic cell types, covering 17,294 genes. Protein expression was measured using mass spectrometry, providing average spectral counts for each protein in normal tissues and cell types. The protein-level spectral data were reported by gene name and were retrieved from the HPM portal in June 2022. The GTEx consortium collected protein expression data from a subset of the RNA-sequenced samples (N=201). Samples were collected and profiled from 14 normal donors and 32 tissue types, covering 12,627 proteins. Tandem mass tag (TMT) mass spectrometry was used to quantify protein expression and identify tissue-enriched and tissue-specific proteins. The log-relative-abundance of proteins and tissue specificity scores were reported by the gene Ensembl ID (Table S2).

*Processing RNA-seq Data from Diseased Tissues:* RNA-seq gene counts from lung tissue (GSE92592) of idiopathic pulmonary fibrosis patients (N=20) compared to healthy controls (N=19) (50), as well as from left ventricular tissue (GSE55296) of patients with heart failure (N=26) compared to healthy controls (N=10) (51) were processed using TargetRanger to identify genes significantly highly expressed in the diseased tissues where senescent cells are known to accumulate.

*Target Identification:* To identify highly expressed genes or transcripts within senescent cells compared to a background dataset, we compared the input expression vectors to expression in non-diseased tissues processed from GTEx (45), ARCHS4 (46), and Tabula Sapiens (52). Each background dataset was processed to extract the 1^st^ quartile, median, and 3^rd^ quartile of each gene and transcript raw counts across all tissues, cell types, and donors. Quantile normalization is used to conform the input samples’ gene expression distribution to that of the background gene expression distribution. Differential expression is performed using limma-voom (53), implemented in Bioconductor (54). The significantly differentially expressed candidates are defined as those with a Benjamini-Hochberg adjusted p-value of less than 0.05 with an additional constraint that the logFC-absolute empirical Bayes moderated t-statistic product is more than 3 standard deviations from the mean. To select genes encoding proteins that are membrane-bound, we retained genes associated with the “cell surface” (GO:0009986) or “plasma membrane” (GO:0005886) categories in the COMPARTMENTS database (55), with a confidence score >=3. These membrane-localized genes were also required to exhibit experimental protein-level evidence and tissue specificity as measured by HPA (56). To select genes encoding extracellular ligands, we also retained genes with predicted secreted products from HPA with protein-level evidence and tissue specificity. Proteomic profiles of each candidate across normal cell types and tissues were obtained from the HPA (47), HPM (48), and GTEx proteomics (49). At the gene level, we analyzed the 13 senescent cell datasets with all three transcriptomics backgrounds for a total of 39 conditions. At the transcript level, the ​​senescent cell datasets were analyzed with the ARCHS4 and GTEx transcriptomics backgrounds (26 conditions). Consistent targets were identified as those appearing in at least 25% of the conditions.

*Enrichment Analysis of Top Candidates:* To uncover molecular pathways and disease associations that may be uniquely highly expressed in RS cells and aged tissues, the top consistent target genes appearing in at least 25 analysis conditions were submitted to Enrichr (57) for enrichment analysis. The results are visualized as a network made of nodes that represent genes and enriched terms using Cytoscape (58).

*The TargetRanger website:* The TargetRanger web-based application was developed using React inside the Next.js framework. Many of the icons and components are sourced from the Material-UI library. The processed data from each resource is stored in a PostgreSQL v14 database and accessed using Prisma. TargetRanger queries the database with expression statistics from the user’s input file and a background dataset and the PostgreSQL database utilizes the SciPy (59) Python library to identify highly expressed targets with a Welch’s t-test. Average expression, standard deviation, and counts across each of the background transcriptomic datasets are computed and stored as a materialized view for efficient access. TargetRanger and its dependencies are containerized using Docker and deployed on a cluster managed by Kubernetes on AWS.

*Comparing SenoRanger to other related sets:* We assembled senescence-related gene sets from LongevityMap (60), CellAge (60), Digital Ageing Atlas (61), GenAge (60), and SenMayo (62). To visualize the similarity between sets we first generated TF-IDF vector representations from each gene set. TF-IDF scores were computed for all genes across the gene sets, with “terms” being gene symbols and “documents” being the eight gene sets, using the TfidfVectorizer from the Python sci-kit learn library. We then applied Uniform Manifold Approximation and Projection (UMAP) (63) to the TF-IDF vectorized gene sets. Scatter plots were generated using the Python bokeh library.

# **Supporting Background**

Cellular senescence is a state of permanent cell cycle arrest that occurs in somatic cells. Cellular senescence is a tightly regulated biological process in normal physiology (1). However, senescence has also been implicated as a key process in aging, where senescent cells avoid clearance by the immune systems and accumulate to contribute to age-related pathologies (2,3). In some mouse tissues such as liver, spleen, skin, and lung it is estimated that the presence of senescence cells increases from ~3-5% to 20-30% (4). Senescent cells undergo distinct morphological and transcriptional changes, adopting a Senescence Associated Secretory Phenotype (SASP) characterized by the secretion of a host of proinflammatory molecules, growth factors, and proteases (5). Various types of stressors can induce senescence, including oncogene activation (6), oxidative stress (7), telomere erosion (8,9), and other sources of DNA damage. Due to such diversity, cell type variability and multiple stages of progression, the phenotypic heterogeneity of senescent cells has made it challenging to identify universal biomarkers of senescence (10,11). Nonetheless, senescent cells appear to have common transcriptional profiles (12), particularly within the same cell types and in response to specific stressors (11).

Replicative senescence (RS) is a specific form of senescence that occurs when cells have reached their maximum capacity to divide (Hayflick’s limit) (13). Triggering RS is dependent on telomere shortening (8), which is related to organism aging (14). RS may have unique molecular contributions to aging that are distinct from other forms of senescence (15); for instance, RS of microglia has been directly linked to Aβ pathology in Alzheimer’s disease (16). Importantly, although mouse models are common in preclinical studies of senescent cells and aging, murine cells do not senesce under the same conditions as human cells due to their longer telomeres and shorter lifespans (17,18).

Whether due to age-related immunodeficiencies (19) or SASP changes (20), senescent cells become increasingly resistant to clearance by immune cells during aging. Senescent cell accumulation has been detected in cardiac disease (21), atherosclerosis (21), lung disease (22,23), diabetes (24), liver disease (25), neurodegenerative disorders (16,26-28), and many other age-associated pathologies. Hence, it was suggested that selective removal of such cell may offer a benefit to prevent or delay the onset of many of these diseases. Indeed, clearance of senescent cells has been demonstrated to delay the onset of aging phenotypes in progeroid mouse models (29) and increased metabolic function (30) and healthy lifespan (3) in naturally-aged mice. In a neurodegenerative mouse model, senescent cell elimination prevented tau pathology (26). Such evidence has led to a rapid growth of interest in developing senolytic therapies that can remove senescent cells in humans. However, current clinical approaches to senescent cell removal remain relatively limited, untested, or nonspecific (2,3,31).

Two promising therapeutic strategies that have demonstrated targeted cell removal *in vivo* is the delivery of cytotoxic drugs using antibody-drug conjugates (ADCs) (32) or chimeric antigen receptor (CAR) T cells (33) that target proteins that are specific to the diseased cells. Both ADC and CAR T cell therapies have been explored as a treatment for a wide range of diseases. However, robust disease cell surface markers are necessary for these treatments to be effective. Evidence suggests that prioritization of cell-surface proteins that are highly expressed in the diseased cells compared to all other normal tissues and cell types can be used to identify effective immunotherapeutic targets. Using this strategy, targets have been identified for the selective removal of cancer cells (34) as well as senescent cells (35-37). For example, Althubiti et al. identified 10 membrane senescence markers using proteomics (35), and demonstrated that targeting one of these markers, namely B2M, with an ADC effectively removed senescent cells without damaging proliferating cells (36). In another study, Amor et al. (37) identified uPAR as a cell-surface protein highly expressed in murine senescent hepatic stellate cells. They confirmed that uPAR was upregulated in tissues from patients with senescence-associated diseases and absent in normal human tissues. CAR T cell-mediated selective elimination of uPAR-positive senescent cells in mice reduced liver fibrosis. While membrane proteins are ideal for targeted senolytic therapies with ADCs or CAR T cells, other approaches can also use non-surface-ome markers to selectively remove senescent cells. For instance, targeting genes with RNA interference (38).

As targeted removal of senescent cells gains more research interest, there is a need for rapid, streamlined screening of transcriptomics and proteomics data that can be used to identify targets for potential removal of various senescent cell types. Here we develop background normal tissue and cell type datasets, algorithms to compute differentially expression, and interactive software to prioritize cell-surface proteins for targeting senescent cells. The results are ranked lists of potential targets for senolytic therapy.

# **Supporting Discussion**

Here we describe a rational method for identifying targets for removal of senescence cells. First, we identified in-vitro studies that characterized senescence cells by various independent laboratories using RNA-seq transcriptomics. Next, we screened for genes and transcripts that are uniquely highly expressed in the senescent cells and lowly expressed across many normal adult cell types and tissues. Specifically, we used three independent backgrounds of normal tissue and cell type signatures to compare to the senescence signatures: GTEx, Tabula Sapiens, and ARCHS4. Consensus potential targets were then filtered by including only those that are membrane proteins, and the genes that are also differentially expressed in old tissues when compared to young tissues from the GTEx cohort. The approach recovered known senescence targets and discovered new potential candidates for targeting senescent cells with immunotherapy. Such novel targets will need to be validated in follow up experiments. Such experiments can begin with the development of several novel ADCs that could be tested first in-vitro for their ability to kill senescence cells followed by experiment in animal models. It is likely that senescence targets in animal models would be different, but if shared candidates are identified that would be preferable.

Before such experiments can begin, it would be best to consider the various limitations of the approach. First, our analysis is applied at the mRNA level while the aim of identifying membrane relevant targets is at the protein level. It is well established that mRNA levels do not correlate well with protein and thus many useful targets may be missed. It is possible that highly ranked targets may not be uniquely expressed at the protein level on the surface of just senescence cells as expected. To mitigate this, the user can check the expression of the gene product against the three proteomics databases that we wrangled. In addition, the 13 RS group of samples are almost all derived from human fibroblasts. It is known that senescence cells across human tissues are very different. Hence, our analysis may miss such heterogeneity. It is also known that senescence has different stages, for example, early vs. deep. We did not account for such differences. Additionally, the expression profiles that we considered in this study are all from senescence induced by replication as opposed to senescence induced by an oncogene, oxidative stress, DNA damage, or other stressors. This means that the targets that we identified may not translate to senescent cells in humans in vivo that were induced by these other stressors. However, we expect that there is some convergence of pathways into one similar phenotype.

The computational pipeline presented in this study also has some limitations. First, the background gene expression data from ARCHS4 was created with tokenized term labeling of RNA-seq samples to categorize ARCHS4 samples into normal tissues and cell types. Such an approach is subject to error both in mislabelling and misleading labels. While the statistics we compute should be somewhat robust to outliers, the presence of strong outliers such as those collected from disease cells may influence the computed normal tissue and cell type backgrounds. In addition, since using all the samples from the background to compute differential expression is impractical, we decided to use quartiles instead. This approach considers a robust distribution for each tissue or cell type, but it is less accurate. The use of differential expression analysis will prioritize highly expressed target candidates, but it does not guarantee that the expression of the target is lowly expressed across all tissues. Moreover, the use of quantile normalization is applied to correct for the distribution differences between the input samples and the preprocessed background in lue of many other options. However, this choice may inflate or deflate key differences. The added logFC-absolute empirical Bayes moderated t-statistic product constraint is likely to be more applicable to a wider range of datasets while the more standard logFC and p-value cutoff using fixed bounds may result in too many or too few hits.

In a prior study, cell surface markers were identified from senescent cells' plasma membrane fraction[^35^](https://www.zotero.org/google-docs/?kRfScf). 107 unique proteins were identified and 10 were further validated. Unfortunately, the lists of identified proteins from this study are not available. The idea of removing senescence cells with an ADC was previously proposed by the same group[^69^](https://www.zotero.org/google-docs/?49sxJE). In their follow up study, the authors showed that targeting B2M with an B2M-duocarmycin ADC can effectively remove senescence cells in-vitro[^36^](https://www.zotero.org/google-docs/?0MHQpz). Altogether, more studies are needed by more independent teams to validate the approach and confirm the value of the targets we identified here. In addition to identifying gene targets, we also identified differentially expressed transcripts. Such transcript isoforms can be targeted by RNAi therapies to disrupt senescent cell physiology toward a phenotype that causes less damage. In summary, here we enlist membrane proteins that are highly expressed in senescent and in aged tissues. These proteins may serve as biomarkers and targets for immunotherapies with senolytic potential.
