# Supplementary material for: Computational screen to identify potential targets for immunotherapeutic identification and removal of senescence cells
Source: Aging Cell. 2023 Apr 20;22(6):e13809. doi: 10.1111/acel.13809 (PMC10265163; doi:10.1111/acel.13809)
Supplement: Supplementary file 2 — Table S2 Top consensus genes identified to be highly expressed in replicative senescence cells compared to normal tissues and cell type backgrounds. [file ACEL-22-e13809-s003.pdf]

Gene	Significant transcript(s)	ARCHS4 (gene level)	GTEx (gene level)	TabulaSapiens (gene level)	Total conditions (gene level)	CellAge	SenMayo	AgedTissue	DiseaseTissue
ALPK2	ENST00000589204	12	12	12	36	0	0	NA	NA
BDNF	ENST00000356660; ENST00000530786; ENST00000533131; ENST00000584049	12	12	12	36	0	0	NA	NA
CPA4	ENST00000222482; ENST00000493259	10	13	13	36	1	0	NA	NA
GALNT5	ENST00000259056	12	12	12	36	1	0	Blood Vessel	NA
TNFRSF11B	ENST00000297350; ENST00000517352; ENST00000521597	12	12	12	36	0	1	Blood Vessel; Brain	NA
FGF5	ENST00000312465; ENST00000456523; ENST00000503413	11	12	12	35	0	0	Nerve	NA
CHRM2	ENST00000401861; ENST00000445907	11	11	11	33	0	0	NA	NA
MMP1	ENST00000315274	12	13	8	33	0	1	NA	Idiopathic Pulmonary Fibrosis
PGAM4	NA	12	12	9	33	0	0	NA	NA
PDCD1LG2	ENST00000397747	12	11	9	32	1	0	Blood Vessel	NA
ADAMTS12	NA	12	7	12	31	0	0	NA	NA
PSG5	ENST00000342951; ENST00000366175	9	12	9	30	1	0	NA	NA
CEMIP	ENST00000220244; ENST00000356249; ENST00000394685; ENST00000495041	9	9	11	29	0	0	Adipose; Blood Vessel	NA
STC2	ENST00000519511	9	8	12	29	1	0	NA	NA
SCN9A	ENST00000303354; ENST00000409435; ENST00000409672; ENST00000454569	10	8	10	28	0	0	NA	NA
WNT5A	ENST00000474267; ENST00000493406	11	5	12	28	1	0	NA	NA
KRTAP1-5	ENST00000361883	10	12	5	27	1	0	NA	NA
LOX	ENST00000231004; ENST00000503759; ENST00000505593; ENST00000508067; ENST00000513319	9	6	12	27	1	0	Lung; Muscle	NA
LRRC17	ENST00000249377; ENST00000339431	9	9	9	27	0	0	Blood Vessel	NA
MARCHF4	ENST00000273067	9	9	9	27	0	0	NA	NA
OXTR	ENST00000316793; ENST00000431493	9	9	9	27	0	0	Blood Vessel; Brain	NA
PAPPA	NA	9	8	10	27	0	1	Nerve	Idiopathic Pulmonary Fibrosis
RAB3B	ENST00000371655	3	12	12	27	0	0	NA	NA
SERPINB7	NA	10	10	7	27	0	0	NA	Idiopathic Pulmonary Fibrosis
TBX5	NA	12	8	7	27	0	0	Lung	NA
COLEC10	ENST00000332843	9	9	8	26	0	0	NA	NA
F2RL2	ENST00000296641	10	10	6	26	0	0	Blood Vessel; Lung; Nerve	Idiopathic Pulmonary Fibrosis
FOXL1	NA	9	8	9	26	0	0	Lung; Muscle	NA
COL11A1	ENST00000512756	9	7	9	25	0	0	Adipose; Blood Vessel	NA
FBN2	NA	4	10	11	25	0	0	Thyroid	NA
GREM1	ENST00000300177	10	3	12	25	0	0	Blood Vessel	Idiopathic Pulmonary Fibrosis

Gene	Significant transcript(s)	ARCHS4 (gene level)	GTEx (gene level)	TabulaSapiens (gene level)	Total conditions (gene level)	CellAge	SenMayo	AgedTissue	DiseaseTissue
LOXL2	ENST00000389131; ENST00000520349	7	5	13	25	1	0	NA	NA
TNFSF4	ENST00000281834; ENST00000367718; ENST00000488053	9	9	7	25	0	0	NA	NA
ZC3H11A	ENST00000367214	0	13	12	25	0	0	NA	NA
COL1A2	ENST00000620463	11	0	12	23	0	0	NA	Idiopathic Pulmonary Fibrosis
DKK3	ENST00000532873; ENST00000534511	11	0	12	23	0	0	NA	NA
FLNC	NA	11	0	12	23	1	0	NA	Idiopathic Pulmonary Fibrosis
GPR176	NA	11	0	12	23	0	0	NA	NA
HMGA2	ENST00000403681; ENST00000541363	0	13	10	23	0	0	NA	NA
PAMR1	ENST00000529303	12	0	11	23	1	0	Blood Vessel; Brain; Lung; Muscle	NA
PSG4	ENST00000244295; ENST00000451895; ENST00000597912; ENST00000601041	6	11	6	23	1	0	NA	NA
RGMB	NA	4	7	12	23	0	0	NA	NA
SPOCD1	ENST00000468720	4	11	8	23	0	0	Blood Vessel; Brain	NA
THY1	ENST00000524970	11	0	12	23	0	0	Blood Vessel	Idiopathic Pulmonary Fibrosis; Heart Failure
TRHDE	ENST00000261180; ENST00000547300; ENST00000549138; ENST00000549922	12	3	8	23	0	0	Lung	NA
BDKRB1	NA	9	8	5	22	0	0	NA	NA
COL12A1	ENST00000416123	10	0	12	22	0	0	NA	NA
FKBP1C	NA	8	8	6	22	0	0	NA	NA
IGFBP5	NA	11	0	11	22	1	1	NA	Idiopathic Pulmonary Fibrosis
ITGBL1	NA	9	7	6	22	0	0	Adipose; Colon; Heart; Lung; Muscle	NA
PCDHGB4	NA	5	10	7	22	0	0	NA	NA
PRSS12	NA	7	7	8	22	0	0	Nerve	NA
SVEP1	NA	11	0	11	22	0	0	Muscle	NA
WNT16	ENST00000222462; ENST00000361301	8	8	6	22	0	1	NA	NA
COL3A1	ENST00000317840	10	0	11	21	0	0	NA	Idiopathic Pulmonary Fibrosis
COL6A3	ENST00000353578	9	0	12	21	0	0	Muscle	Idiopathic Pulmonary Fibrosis
IGF2BP3	ENST00000258729; ENST00000619562	0	12	9	21	0	0	NA	NA
PTCHD4	NA	7	10	4	21	0	0	Adipose; Blood Vessel; Lung; Nerve; Thyroid	NA
TRPA1	ENST00000262209; ENST00000520596; ENST00000522271; ENST00000523582	7	7	7	21	0	0	NA	NA
COL6A2	NA	10	0	10	20	0	0	NA	NA
DKK1	NA	5	10	5	20	0	1	Blood Vessel	NA
GFRA1	NA	11	0	9	20	0	0	NA	NA
IGF2BP1	ENST00000290341	0	11	9	20	0	0	NA	NA
MGARP	ENST00000398955	10	9	1	20	1	0	Adipose; Blood Vessel; Heart	NA
NCKAP5	NA	10	6	4	20	1	0	Blood Vessel	NA
NID2	NA	9	1	10	20	0	0	NA	NA
NOMO3	NA	0	10	10	20	0	0	NA	NA

Gene	Significant transcript(s)	ARCHS4 (gene level)	GTEx (gene level)	TabulaSapiens (gene level)	Total conditions (gene level)	CellAge	SenMayo	AgedTissue	DiseaseTissue
PDGFRA	ENST00000509490	10	0	10	20	0	0	NA	NA
PSG1	NA	8	10	2	20	1	0	NA	NA
RSPO1	NA	7	6	7	20	0	0	NA	NA
SERPINE1	NA	8	0	12	20	0	1	Blood Vessel; Brain	NA
WNT5B	NA	12	0	8	20	0	0	NA	NA
ANO4	ENST00000392977	9	3	7	19	0	0	NA	NA
COL1A1	ENST00000474644	8	0	11	19	0	0	Brain	Idiopathic Pulmonary Fibrosis
COL5A2	NA	7	0	12	19	0	0	NA	Idiopathic Pulmonary Fibrosis
CPED1	ENST00000443817	12	0	7	19	0	0	Lung	NA
FOXF1	ENST00000262426	12	0	7	19	0	0	Brain	NA
LIF	NA	7	6	6	19	0	0	Adrenal Gland	NA
MAGED4B	NA	0	11	8	19	0	0	NA	NA
PABPC3	NA	9	9	1	19	0	0	NA	NA
PCDH18	ENST00000510305	10	0	9	19	0	0	NA	NA
RAB6C	NA	9	9	1	19	0	0	NA	NA
BDKRB2	NA	10	0	8	18	0	0	Muscle	NA
CSNK2A3	NA	7	11	0	18	0	0	NA	NA
DNAH5	NA	7	7	4	18	0	0	NA	NA
EMILIN1	NA	10	0	8	18	0	0	Brain	NA
FMN2	NA	9	0	9	18	0	0	Adipose	NA
GAS6	ENST00000480426; ENST00000610073	10	0	8	18	1	0	Blood Vessel	NA
H3-5	NA	8	9	1	18	0	0	NA	NA
IRAG1	NA	11	0	7	18	0	0	NA	NA
JCAD	NA	9	0	9	18	0	0	NA	NA
MAGED4	ENST00000497348	0	10	8	18	0	0	NA	NA
MASP1	NA	9	0	9	18	1	0	Blood Vessel; Lung	NA
PDE5A	ENST00000264805; ENST00000394439	9	0	9	18	0	0	NA	NA
PLAT	ENST00000352041; ENST00000519510	9	0	9	18	1	1	Blood Vessel; Muscle	NA
PLAU	NA	7	3	8	18	0	1	NA	NA
SERPINB2	NA	7	9	2	18	1	0	NA	NA
SFRP1	NA	9	0	9	18	0	0	Muscle	NA
SGIP1	ENST00000435165	11	0	7	18	1	0	NA	NA
ARL17A	NA	9	7	1	17	0	0	NA	NA
CCN2	NA	8	0	9	17	0	0	NA	NA
EDA2R	NA	10	0	7	17	0	0	Adipose; Adrenal Gland; Blood Vessel; Brain; Colon; Heart; Lung; Muscle; Nerve; Pancreas; Thyroid	NA
EDIL3	NA	8	0	9	17	1	0	Heart; Muscle	NA
FBN1	ENST00000537463	5	0	12	17	0	0	NA	Idiopathic Pulmonary Fibrosis
FN1	ENST00000354785; ENST00000356005; ENST00000426059; ENST00000432072; ENST00000443816; ENST00000446046	5	0	12	17	1	0	NA	NA
FRMD6	ENST00000344768; ENST00000554167	5	0	12	17	1	0	NA	NA
KRT34	NA	5	7	5	17	0	0	NA	NA
LTBP1	ENST00000402934; ENST00000407925; ENST00000422669	8	0	9	17	0	0	NA	Idiopathic Pulmonary Fibrosis

Gene	Significant transcript(s)	ARCHS4 (gene level)	GTEx (gene level)	TabulaSapiens (gene level)	Total conditions (gene level)	CellAge	SenMayo	AgedTissue	DiseaseTissue
MYLK	ENST00000360304	6	0	11	17	1	0	NA	NA
NLRP10	NA	4	11	2	17	0	0	NA	NA
PSG6	NA	4	12	1	17	1	0	NA	NA
STC1	NA	10	0	7	17	1	0	Blood Vessel; Heart; Muscle	NA
TMEM47	NA	10	0	7	17	1	0	NA	NA
TNFRSF10D	NA	6	5	6	17	1	0	Brain	NA
TNFSF18	ENST00000404377	7	7	3	17	0	0	NA	NA
ACTG2	NA	9	0	7	16	0	0	NA	Idiopathic Pulmonary Fibrosis
ADGRA2	NA	8	0	8	16	0	0	NA	NA
CD248	NA	9	0	7	16	0	0	NA	NA
CLDN1	NA	9	0	7	16	1	0	Blood Vessel	NA
CNN1	ENST00000592338	11	0	5	16	1	0	NA	NA
COL5A1	NA	5	0	11	16	0	0	NA	Idiopathic Pulmonary Fibrosis
CTHRC1	ENST00000520337	8	5	3	16	0	0	Adipose; Blood Vessel; Muscle	Idiopathic Pulmonary Fibrosis
DCBLD2	ENST00000326857	4	0	12	16	0	0	NA	NA
DCN	ENST00000420120	8	0	8	16	0	0	Muscle	NA
ITGA11	NA	6	0	10	16	0	0	Blood Vessel; Brain; Colon; Heart; Lung	NA
KISS1	NA	6	7	3	16	0	0	NA	NA
LUM	NA	8	0	8	16	1	0	Adipose; Blood Vessel; Muscle	NA
MMP2	ENST00000543485; ENST00000564864	7	0	9	16	0	1	Lung; Muscle	Idiopathic Pulmonary Fibrosis
MOXD1	NA	8	0	8	16	0	0	Blood Vessel; Nerve	Idiopathic Pulmonary Fibrosis
MXRA5	NA	8	0	8	16	0	0	Blood Vessel	NA
MYPN	NA	6	6	4	16	0	0	NA	NA
NES	NA	8	0	8	16	0	0	NA	NA
NPTX1	NA	7	2	7	16	0	0	Lung	NA
PDE1C	NA	8	0	8	16	0	0	Adipose	NA
PTGS1	NA	10	0	6	16	0	0	NA	NA
RAB6D	NA	8	8	0	16	0	0	NA	NA
TMTC1	ENST00000539277	9	0	7	16	1	0	NA	NA
ARSJ	ENST00000509829	8	0	7	15	0	0	NA	NA
COL16A1	NA	5	0	10	15	0	0	Blood Vessel; Heart	NA
CYB5D1	NA	0	13	2	15	0	0	NA	NA
FIBIN	NA	10	0	5	15	0	0	Adipose; Colon; Heart; Muscle	NA
FKBP10	NA	3	0	12	15	0	0	NA	NA
LPAR1	ENST00000541779	7	0	8	15	0	0	NA	NA
LRRC15	NA	6	6	3	15	0	0	Blood Vessel	NA
MMP3	NA	6	6	3	15	1	1	NA	Idiopathic Pulmonary Fibrosis
NT5E	ENST00000416334	6	0	9	15	0	0	Muscle	NA
SGCD	NA	9	0	6	15	0	0	NA	NA
SH2D5	NA	8	0	7	15	0	0	NA	NA
TEK	ENST00000406359	9	0	6	15	0	0	NA	NA
THBS1	ENST00000260356; ENST00000397591; ENST00000497720	4	0	11	15	1	0	Blood Vessel	NA
UCHL1	NA	3	0	12	15	1	0	NA	NA
CCDC68	ENST00000337363	8	3	3	14	0	0	Blood Vessel	NA
CDK15	NA	10	4	0	14	0	0	NA	NA
CNTNAP1	NA	5	0	9	14	0	0	NA	NA
COL7A1	NA	5	0	9	14	0	0	Lung; Muscle; Nerve	NA
CREB3L1	NA	7	0	7	14	0	0	NA	NA
CSPG4	NA	6	0	8	14	0	0	NA	NA
DDX47	NA	0	9	5	14	0	0	NA	NA

Gene	Significant transcript(s)	ARCHS4 (gene level)	GTEx (gene level)	TabulaSapiens (gene level)	Total conditions (gene level)	CellAge	SenMayo	AgedTissue	DiseaseTissue
ECM1	ENST00000369049	7	0	7	14	0	0	Blood Vessel	NA
FZD7	NA	7	0	7	14	0	0	NA	NA
GLUD2	NA	9	4	1	14	0	0	NA	NA
LAYN	NA	11	0	3	14	0	0	Colon	NA
LOXL4	ENST00000260702	7	2	5	14	0	0	Colon; Muscle	NA
PODN	NA	10	0	4	14	0	0	Muscle	NA
GES3L-AARS	NA	8	0	6	14	0	0	NA	NA
PXDN	ENST00000453308	2	0	12	14	0	0	NA	NA
RGPD6	NA	0	12	2	14	0	0	NA	NA
SEMA3A	ENST00000436949	3	2	9	14	0	0	Blood Vessel	NA
TGFBI	ENST00000507018	4	0	10	14	0	0	NA	NA
TMEM178B	NA	7	0	7	14	1	0	Nerve	NA
ADAM33	ENST00000350009	8	0	5	13	0	0	Adipose; Brain; Colon; Lung	NA
ANGPTL2	NA	7	0	6	13	0	0	NA	NA
ARL2BP	ENST00000563234	0	13	0	13	0	0	NA	NA
CDH11	NA	5	0	8	13	0	0	Blood Vessel; Muscle	NA
DPP4	ENST00000490286	9	2	2	13	1	0	Adipose; Blood Vessel; Muscle	NA
EIF2S3B	NA	6	7	0	13	0	0	NA	NA
INHBA	NA	4	5	4	13	0	0	NA	NA
LMOD1	NA	12	0	1	13	1	0	Colon; Lung	NA
MXRA8	NA	5	0	8	13	0	0	NA	NA
PDGFRB	NA	8	0	5	13	0	0	NA	NA
PSG7	NA	1	12	0	13	1	0	NA	NA
PSG8	NA	3	9	1	13	0	0	NA	NA
PTP4A1	NA	0	0	13	13	0	0	NA	NA
SMIM11	NA	1	11	1	13	0	0	NA	NA
TBX2	NA	8	0	5	13	0	0	Brain	NA
TMEM158	NA	9	0	4	13	0	0	NA	NA
TOP3B	NA	0	13	0	13	0	0	NA	NA
ZBED6	NA	0	11	2	13	0	0	NA	NA
ZBTB9	NA	0	13	0	13	0	0	NA	NA
ZSCAN32	NA	0	13	0	13	0	0	NA	NA
ADAMTS19	ENST00000274487	5	4	3	12	0	0	NA	NA
ADAMTS5	NA	10	0	2	12	1	0	NA	NA
ANTXR1	NA	0	0	12	12	1	0	NA	NA
ATG9A	NA	0	0	12	12	0	0	NA	NA
CALU	ENST00000493278	0	0	12	12	0	0	NA	NA
CCND1	NA	1	0	11	12	1	0	NA	NA
CD99	NA	0	0	12	12	0	0	Brain	NA
DDAH1	NA	0	0	12	12	1	0	NA	NA
EVC	NA	0	0	12	12	0	0	NA	NA
FAT1	ENST00000509647; ENST00000614102	0	0	12	12	1	0	NA	NA
FST	NA	5	0	7	12	0	0	Blood Vessel; Lung; Muscle	NA
FSTL1	ENST00000424703; ENST00000469005	0	0	12	12	0	0	NA	NA
GJD3	NA	0	12	0	12	0	0	NA	NA
GPX8	NA	0	0	12	12	0	0	Muscle	NA
H3C14	NA	1	10	1	12	0	0	NA	NA
H3C15	NA	1	10	1	12	0	0	NA	NA
HLA-G	NA	8	4	0	12	0	0	NA	NA
IGFBP3	NA	6	0	6	12	1	1	Colon; Heart; Lung	Heart Failure
IGFBP4	NA	6	0	6	12	0	1	NA	NA
KIRREL1	NA	0	0	12	12	0	0	NA	NA

Gene	Significant transcript(s)	ARCHS4 (gene level)	GTEx (gene level)	TabulaSapiens (gene level)	Total conditions (gene level)	CellAge	SenMayo	AgedTissue	DiseaseTissue
KRTAP2-2	NA	2	9	1	12	0	0	NA	NA
MATR3	ENST00000510056; ENST00000512040	0	11	1	12	0	0	NA	NA
NTF3	NA	12	0	0	12	0	0	Lung	NA
POLR2J2	NA	0	12	0	12	0	0	NA	NA
PSG2	NA	0	12	0	12	1	0	NA	NA
RAI14	NA	0	0	12	12	1	0	NA	NA
RELN	NA	7	0	5	12	0	0	Muscle	NA
RGPD3	NA	2	9	1	12	0	0	NA	NA
RGPD5	NA	0	12	0	12	0	0	NA	NA
PL17-C18orf3	ENST00000577910	2	0	10	12	0	0	NA	NA
SPARC	ENST00000539687	0	0	12	12	1	0	NA	NA
TAGLN	NA	7	0	5	12	0	0	NA	NA
ZNF593	NA	0	12	0	12	0	0	NA	NA
ACKR4	ENST00000249887	5	3	3	11	0	0	NA	NA
ANGPT1	ENST00000520052	8	0	3	11	1	1	NA	NA
ATP5MGL	NA	1	10	0	11	0	0	NA	NA
CCDC80	ENST00000475181	1	0	10	11	0	0	NA	NA
CCDC9B	NA	1	0	10	11	1	0	NA	NA
CDH2	NA	3	0	8	11	0	0	Blood Vessel; Nerve	Idiopathic Pulmonary Fibrosis
CHRNA1	NA	4	7	0	11	0	0	NA	NA
COL4A5	NA	2	0	9	11	0	0	NA	NA
ORO7-PAM1	NA	9	0	2	11	0	0	NA	NA
E2F7	NA	0	9	2	11	0	0	NA	NA
EFEMP1	NA	3	0	8	11	1	0	Brain; Muscle	NA
FBLN5	NA	8	0	3	11	0	0	Lung	NA
FZD2	NA	5	6	0	11	0	0	NA	NA
GATD3	NA	0	11	0	11	0	0	NA	NA
GDF6	NA	4	4	3	11	0	0	Adipose; Heart	NA
IL11	NA	3	7	1	11	0	0	NA	NA
IL12A	NA	5	5	1	11	0	0	NA	NA
ITGA2	ENST00000509814; ENST00000513685	3	1	7	11	1	1	NA	NA
KRTAP2-3	NA	0	11	0	11	0	0	NA	NA
LAMC1	ENST00000478064; ENST00000479499	0	0	11	11	1	0	NA	NA
MRGPRF	NA	11	0	0	11	0	0	NA	NA
NALF1	NA	6	0	5	11	0	0	NA	NA
NTM	ENST00000425719	6	0	5	11	0	0	Colon; Nerve	NA
NUDT4B	NA	0	11	0	11	0	0	NA	NA
P3H2	NA	4	0	7	11	1	0	NA	NA
P3H3	NA	3	0	8	11	0	0	NA	NA
PITX1	NA	4	3	4	11	0	0	NA	Idiopathic Pulmonary Fibrosis
PPP1R3C	NA	5	0	6	11	1	0	NA	NA
PSG3	NA	1	10	0	11	0	0	NA	NA
PSG9	ENST00000621109	0	11	0	11	1	0	NA	NA
RND3	ENST00000454202; ENST00000497865	4	0	7	11	1	0	NA	NA
SEMA5A	NA	1	0	10	11	0	0	NA	NA
SERPINE2	ENST00000478966	2	0	9	11	0	1	NA	Idiopathic Pulmonary Fibrosis; Heart Failure
SYNC	NA	5	6	0	11	0	0	NA	NA
TFPI2	NA	5	4	2	11	0	0	NA	NA
THBS2	ENST00000617924	5	0	6	11	0	0	Blood Vessel	Idiopathic Pulmonary Fibrosis

Gene	Significant transcript(s)	ARCHS4 (gene level)	GTEx (gene level)	TabulaSapiens (gene level)	Total conditions (gene level)	CellAge	SenMayo	AgedTissue	DiseaseTissue
THSD4	NA	1	0	10	11	0	0	NA	NA
TPM2	ENST00000604975	1	0	10	11	1	0	Colon	NA
VAMP7	NA	0	0	11	11	0	0	NA	NA
ADAMTS8	NA	7	0	3	10	0	0	Nerve	NA
AJUBA	NA	0	0	10	10	0	0	NA	NA
ALDH1L2	NA	3	0	7	10	0	0	NA	NA
AMIGO3	NA	2	6	2	10	0	0	NA	NA
ANKRD1	NA	3	3	4	10	1	0	Blood Vessel	Heart Failure
CALD1	ENST00000430085	0	0	10	10	1	0	NA	NA
CERCAM	ENST00000420512; ENST00000612334	0	0	10	10	1	0	NA	NA
COL15A1	NA	5	0	5	10	0	0	Muscle	Idiopathic Pulmonary Fibrosis
COL8A1	ENST00000273342	1	0	9	10	1	0	Colon	NA
COPZ2	NA	10	0	0	10	0	0	NA	NA
CSDC2	NA	9	0	1	10	0	0	Lung	NA
EFEMP2	ENST00000527378	3	0	7	10	0	0	NA	NA
EIF3C	NA	0	9	1	10	0	0	NA	NA
EIF5AL1	NA	1	8	1	10	0	0	NA	NA
ERCC5	NA	0	10	0	10	0	0	NA	NA
FAM156B	NA	0	10	0	10	0	0	NA	NA
FBLN2	NA	5	0	5	10	0	0	Heart; Muscle	Idiopathic Pulmonary Fibrosis
FOXD1	NA	5	5	0	10	0	0	NA	NA
FOXF2	NA	9	0	1	10	0	0	NA	NA
GNG5B	NA	3	7	0	10	0	0	NA	NA
GPC1	ENST00000455111	0	0	10	10	1	0	NA	NA
IL6	NA	7	3	0	10	0	1	Brain	NA
LAMA1	ENST00000579014	2	2	6	10	0	0	NA	NA
PTK7	ENST00000352931; ENST00000470019; ENST00000481273	0	0	10	10	0	0	Blood Vessel	NA
PWP2	NA	0	10	0	10	0	0	NA	NA
QSOX1	ENST00000392029	1	0	9	10	0	0	NA	NA
RAPH1	NA	0	10	0	10	0	0	NA	NA
RECK	NA	8	0	2	10	0	0	NA	NA
SERF1A	NA	0	10	0	10	0	0	NA	NA
SMURF2	ENST00000262435; ENST00000585301	2	4	4	10	1	0	NA	NA
SULF1	ENST00000419716; ENST00000616868	5	0	5	10	1	0	NA	Idiopathic Pulmonary Fibrosis
TGFB2	NA	2	2	6	10	1	0	NA	NA
TMEM200A	NA	6	0	4	10	0	0	Blood Vessel	NA
TMEM262	NA	0	10	0	10	0	0	NA	NA
U2AF1	NA	0	10	0	10	0	0	NA	NA
ULBP3	NA	2	8	0	10	0	0	NA	NA
VEPH1	ENST00000392832	3	5	2	10	0	0	NA	NA
WFDC1	NA	5	0	5	10	0	0	NA	NA
ZC3H11C	NA	0	10	0	10	0	0	NA	NA
