# Supplementary material for: Computational screen to identify potential targets for immunotherapeutic identification and removal of senescence cells
Source: Aging Cell. 2023 Apr 20;22(6):e13809. doi: 10.1111/acel.13809 (PMC10265163; doi:10.1111/acel.13809)
Supplement: Supplementary file 3 — Table S3 Top consensus membrane proteins identified to be highly expressed in replicative senescence cells compared to normal tissues and cell type backgrounds. [file ACEL-22-e13809-s004.pdf]

Gene	Significant transcript(s)	ARCHS4 (gene level)	GTEx (gene level)	TabulaSapiens (gene level)	Total conditions (gene level)	CellAge	SenMayo	AgedTissue	DiseaseTissue
CHRM2	ENST00000401861; ENST00000445907	11	11	11	33	0	0	NA	NA
PDCD1LG2	ENST00000397747	12	11	9	32	1	0	Blood.Vessel	NA
SCN9A	ENST00000303354; ENST00000409435; ENST00000409672; ENST00000454569	10	8	10	28	0	0	NA	NA
OXTR	ENST00000316793; ENST00000431493	9	9	9	27	0	0	Blood.Vessel; Brain	NA
F2RL2	ENST00000296641	10	10	6	26	0	0	Blood.Vessel; Lung; Nerve	Idiopathic.Pulmonary.Fibrosis
THY1	ENST00000524970	11	0	12	23	0	0	Blood.Vessel	Idiopathic.Pulmonary.Fibrosis; Heart.Failure
TRHDE	ENST00000261180; ENST00000547300; ENST00000549138; ENST00000549922	12	3	8	23	0	0	Lung	NA
BDKRB1	NA	9	8	5	22	0	0	NA	NA
TRPA1	ENST00000262209; ENST00000520596; ENST00000522271; ENST00000523582	7	7	7	21	0	0	NA	NA
PDGFRA	ENST00000509490	10	0	10	20	0	0	NA	NA
ANO4	ENST00000392977	9	3	7	19	0	0	NA	NA
PCDH18	ENST00000510305	10	0	9	19	0	0	NA	NA
BDKRB2	NA	10	0	8	18	0	0	Muscle	NA
MYLK	ENST00000360304	6	0	11	17	1	0	NA	NA
CD248	NA	9	0	7	16	0	0	NA	NA
CLDN1	NA	9	0	7	16	1	0	Blood.Vessel	NA
ITGA11	NA	6	0	10	16	0	0	Blood.Vessel; Brain; Colon; Heart; Lung	NA
LPAR1	ENST00000541779	7	0	8	15	0	0	NA	NA
NT5E	ENST00000416334	6	0	9	15	0	0	Muscle	NA
SGCD	NA	9	0	6	15	0	0	NA	NA
TEK	ENST00000406359	9	0	6	15	0	0	NA	NA
CNTNAP1	NA	5	0	9	14	0	0	NA	NA
CSPG4	NA	6	0	8	14	0	0	NA	NA
CDH11	NA	5	0	8	13	0	0	Blood.Vessel; Muscle	NA
FAT1	ENST00000509647; ENST00000614102	0	0	12	12	1	0	NA	NA
GJD3	NA	0	12	0	12	0	0	NA	NA
HLA-G	NA	8	4	0	12	0	0	NA	NA
ACKR4	ENST00000249887	5	3	3	11	0	0	NA	NA
CDH2	NA	3	0	8	11	0	0	Blood.Vessel; Nerve	Idiopathic.Pulmonary.Fibrosis
CHRNA1	NA	4	7	0	11	0	0	NA	NA
FZD2	NA	5	6	0	11	0	0	NA	NA
IL12A	NA	5	5	1	11	0	0	NA	NA
MRGPRF	NA	11	0	0	11	0	0	NA	NA
NTM	ENST00000425719	6	0	5	11	0	0	Colon; Nerve	NA
PSG9	ENST00000621109	0	11	0	11	1	0	NA	NA
SEMA5A	NA	1	0	10	11	0	0	NA	NA
GPC1	ENST00000455111	0	0	10	10	1	0	NA	NA
TMEM262	NA	0	10	0	10	0	0	NA	NA
