# Supplementary material for: Computational screen to identify potential targets for immunotherapeutic identification and removal of senescence cells
Source: Aging Cell. 2023 Apr 20;22(6):e13809. doi: 10.1111/acel.13809 (PMC10265163; doi:10.1111/acel.13809)
Supplement: Supplementary file 4 — Table S4 Top consensus secreted proteins identified to be highly expressed in replicative senescence cells compared to normal tissues and cell type backgrounds. [file ACEL-22-e13809-s002.pdf]

Gene	Significant transcript(s)	ARCHS4 (gene level)	GTEx (gene level)	TabulaSapiens (gene level)	Total conditions (gene level)	CellAge	SenMayo	AgedTissue	DiseaseTissue
BDNF	ENST00000356660; ENST00000530786; ENST00000533131; ENST00000584049	12	12	12	36	0	0	NA	NA
CPA4	ENST00000222482; ENST00000493259	10	13	13	36	1	0	NA	NA
TNFRSF11B	ENST00000297350; ENST00000517352; ENST00000521597	12	12	12	36	0	1	Blood.Vessel; Brain	NA
FGF5	ENST00000312465; ENST00000456523; ENST00000503413	11	12	12	35	0	0	Nerve	NA
MMP1	ENST00000315274	12	13	8	33	0	1	NA	Idiopathic.Pulmonary.Fibrosis
PDCD1LG2	ENST00000397747	12	11	9	32	1	0	Blood.Vessel	NA
PSG5	ENST00000342951; ENST00000366175	9	12	9	30	1	0	NA	NA
CEMIP	ENST00000220244; ENST00000356249; ENST00000394685; ENST00000495041	9	9	11	29	0	0	Adipose; Blood.Vessel	NA
STC2	ENST00000519511	9	8	12	29	1	0	NA	NA
WNT5A	ENST00000474267; ENST00000493406	11	5	12	28	1	0	NA	NA
LOX	ENST00000231004; ENST00000503759; ENST00000505593; ENST00000508067; ENST00000513319	9	6	12	27	1	0	Lung; Muscle	NA
LRRC17	ENST00000249377; ENST00000339431	9	9	9	27	0	0	Blood.Vessel	NA
PAPPA	NA	9	8	10	27	0	1	Nerve	Idiopathic.Pulmonary.Fibrosis
COLEC10	ENST00000332843	9	9	8	26	0	0	NA	NA
COL11A1	ENST00000512756	9	7	9	25	0	0	Adipose; Blood.Vessel	NA
FBN2	NA	4	10	11	25	0	0	Thyroid	NA
GREM1	ENST00000300177	10	3	12	25	0	0	Blood.Vessel	Idiopathic.Pulmonary.Fibrosis
LOXL2	ENST00000389131; ENST00000520349	7	5	13	25	1	0	NA	NA
COL1A2	ENST00000620463	11	0	12	23	0	0	NA	Idiopathic.Pulmonary.Fibrosis
DKK3	ENST00000532873; ENST00000534511	11	0	12	23	0	0	NA	NA
PSG4	ENST00000244295; ENST00000451895; ENST00000597912; ENST00000601041	6	11	6	23	1	0	NA	NA
IGFBP5	NA	11	0	11	22	1	1	NA	Idiopathic.Pulmonary.Fibrosis

Gene	Significant transcript(s)	ARCHS4 (gene level)	GTEx (gene level)	TabulaSapiens (gene level)	Total conditions (gene level)	CellAge	SenMayo	AgedTissue	DiseaseTissue
WNT16	ENST00000222462; ENST00000361301	8	8	6	22	0	1	NA	NA
COL3A1	ENST00000317840	10	0	11	21	0	0	NA	Idiopathic.Pulmonary.Fibrosis
COL6A3	ENST00000353578	9	0	12	21	0	0	Muscle	Idiopathic.Pulmonary.Fibrosis
DKK1	NA	5	10	5	20	0	1	Blood.Vessel	NA
NID2	NA	9	1	10	20	0	0	NA	NA
PSG1	NA	8	10	2	20	1	0	NA	NA
RSPO1	NA	7	6	7	20	0	0	NA	NA
SERPINE1	NA	8	0	12	20	0	1	Blood.Vessel; Brain	NA
WNT5B	NA	12	0	8	20	0	0	NA	NA
COL1A1	ENST00000474644	8	0	11	19	0	0	Brain	Idiopathic.Pulmonary.Fibrosis
LIF	NA	7	6	6	19	0	0	Adrenal.Gland	NA
MASP1	NA	9	0	9	18	1	0	Blood.Vessel; Lung	NA
PLAT	ENST00000352041; ENST00000519510	9	0	9	18	1	1	Blood.Vessel; Muscle	NA
PLAU	NA	7	3	8	18	0	1	NA	NA
SFRP1	NA	9	0	9	18	0	0	Muscle	NA
EDIL3	NA	8	0	9	17	1	0	Heart; Muscle	NA
FBN1	ENST00000537463	5	0	12	17	0	0	NA	Idiopathic.Pulmonary.Fibrosis
FN1	ENST00000354785; ENST00000356005; ENST00000426059; ENST00000432072; ENST00000443816; ENST00000446046	5	0	12	17	1	0	NA	NA
LTBP1	ENST00000402934; ENST00000407925; ENST00000422669	8	0	9	17	0	0	NA	Idiopathic.Pulmonary.Fibrosis
PSG6	NA	4	12	1	17	1	0	NA	NA
STC1	NA	10	0	7	17	1	0	Blood.Vessel; Heart; Muscle	NA
COL5A1	NA	5	0	11	16	0	0	NA	Idiopathic.Pulmonary.Fibrosis
CTHRC1	ENST00000520337	8	5	3	16	0	0	Adipose; Blood.Vessel; Muscle	Idiopathic.Pulmonary.Fibrosis
KISS1	NA	6	7	3	16	0	0	NA	NA
LUM	NA	8	0	8	16	1	0	Adipose; Blood.Vessel; Muscle	NA
MMP2	ENST00000543485; ENST00000564864	7	0	9	16	0	1	Lung; Muscle	Idiopathic.Pulmonary.Fibrosis
MXRA5	NA	8	0	8	16	0	0	Blood.Vessel	NA
NPTX1	NA	7	2	7	16	0	0	Lung	NA
MMP3	NA	6	6	3	15	1	1	NA	Idiopathic.Pulmonary.Fibrosis
TEK	ENST00000406359	9	0	6	15	0	0	NA	NA
COL7A1	NA	5	0	9	14	0	0	Lung; Muscle; Nerve	NA

Gene	Significant transcript(s)	ARCHS4 (gene level)	GTEx (gene level)	TabulaSapiens (gene level)	Total conditions (gene level)	CellAge	SenMayo	AgedTissue	DiseaseTissue
ECM1	ENST00000369049	7	0	7	14	0	0	Blood.Vessel	NA
SEMA3A	ENST00000436949	3	2	9	14	0	0	Blood.Vessel	NA
TGFBI	ENST00000507018	4	0	10	14	0	0	NA	NA
DPP4	ENST00000490286	9	2	2	13	1	0	Adipose; Blood.Vessel; Muscle	NA
INHBA	NA	4	5	4	13	0	0	NA	NA
PSG7	NA	1	12	0	13	1	0	NA	NA
PSG8	NA	3	9	1	13	0	0	NA	NA
ADAMTS5	NA	10	0	2	12	1	0	NA	NA
FST	NA	5	0	7	12	0	0	Blood.Vessel; Lung; Muscle	NA
HLA-G	NA	8	4	0	12	0	0	NA	NA
IGFBP3	NA	6	0	6	12	1	1	Colon; Heart; Lung	Heart.Failure
PSG2	NA	0	12	0	12	1	0	NA	NA
RELN	NA	7	0	5	12	0	0	Muscle	NA
SPARC	ENST00000539687	0	0	12	12	1	0	NA	NA
ANGPT1	ENST00000520052	8	0	3	11	1	1	NA	NA
COL4A5	NA	2	0	9	11	0	0	NA	NA
GDF6	NA	4	4	3	11	0	0	Adipose; Heart	NA
IL12A	NA	5	5	1	11	0	0	NA	NA
LAMC1	ENST00000478064; ENST00000479499	0	0	11	11	1	0	NA	NA
PSG3	NA	1	10	0	11	0	0	NA	NA
PSG9	ENST00000621109	0	11	0	11	1	0	NA	NA
SERPINE2	ENST00000478966	2	0	9	11	0	1	NA	Idiopathic.Pulmonary.Fibrosis; Heart.Failure
TFPI2	NA	5	4	2	11	0	0	NA	NA
THSD4	NA	1	0	10	11	0	0	NA	NA
ADAMTS8	NA	7	0	3	10	0	0	Nerve	NA
COL15A1	NA	5	0	5	10	0	0	Muscle	Idiopathic.Pulmonary.Fibrosis
FBLN2	NA	5	0	5	10	0	0	Heart; Muscle	Idiopathic.Pulmonary.Fibrosis
IL6	NA	7	3	0	10	0	1	Brain	NA
LAMA1	ENST00000579014	2	2	6	10	0	0	NA	NA
WFDC1	NA	5	0	5	10	0	0	NA	NA
